# uPAR: An Essential Factor for Tumor Development

**DOI:** 10.7150/jca.62281

**Published:** 2021-10-17

**Authors:** Tao Lv, Ying Zhao, Xinni Jiang, Hemei Yuan, Haibo Wang, Xuelin Cui, Jiashun Xu, Jingye Zhao, Jianlin Wang

**Affiliations:** 1 College of Biological Resource and Food Engineering, Qujing Normal University, Qujing, Yunnan, China 655011.; 2 Key Laboratory of Yunnan Province Universities of the Diversity and Ecological Adaptive Evolution for Animals and Plants on YunGui Plateau, Qujing Normal University, Qujing, China 655011.; 3 School of Biological Sciences and Technology, Chengdu Medical College, Chengdu, Sichuan, China 610500.; 4 College of Chemistry and Environmental Science, Qujing Normal University, Qujing, Yunnan, China 655011.

**Keywords:** uPAR, tumorigenesis, proliferation, adhesion, metastasis, cancer therapy

## Abstract

Tumorigenesis is closely related to the loss of control of many genes. Urokinase-type plasminogen activator receptor (uPAR), a glycolipid-anchored protein on the cell surface, is controlled by many factors in tumorigenesis and is expressed in many tumor tissues. In this review, we summarize the regulatory effects of the uPAR signaling pathway on processes and factors related to tumor progression, such as tumor cell proliferation, adhesion, metastasis, glycolysis, tumor microenvironment and angiogenesis. Overall, the evidence accumulated to date suggests that uPAR induction by tumor progression may be one of the most important factors affecting therapeutic efficacy. An improved understanding of the interactions between uPAR and its coreceptors in cancer will provide critical biomolecular information that may help to better predict the disease course and response to therapy.

## Introduction

Tumors are the result of uncontrolled proliferation of cells in different organs. Tumor development is a multistage process, that includes the generation of primary tumors, separation of tumors from primary sites, degradation of extracellular matrix (ECM), and distant metastasis of tumors. A variety of genes play important roles in the development of tumors 1-3, including the cell surface receptor urokinase-type plasminogen activator receptor (uPAR). uPAR is highly expressed in a variety of tumor cells, and a variety of signals regulated by uPAR play significant roles in tumor cell proliferation and metastasis, tumor-related glycolysis, the tumor microenvironment and angiogenesis 4-6. Studies have found that some specific drugs and antibodies have unique inhibitory effects on uPAR. This review intends to deliver an overview of current knowledge about the role of uPAR in cancer progression and attempts to provide a theoretical basis for tumor therapy.

## Structural characteristics of uPAR

**Figure 1:**
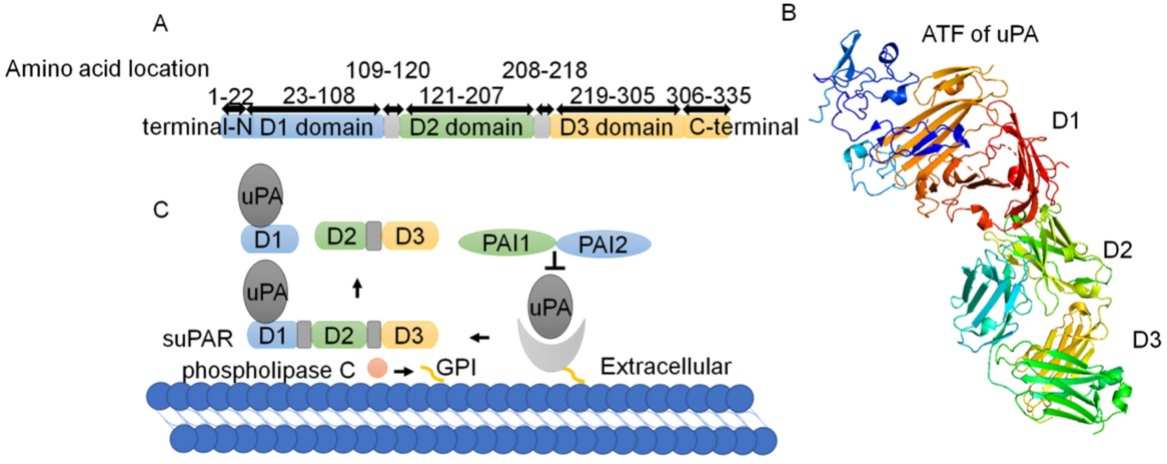
** Two dimensional (2D) and three dimensional (3D) structures of uPAR. (A)** uPAR encodes a protein of 335 amino acids comprising 22 amino acids (secreted signal peptides) at the N-terminus and 30 amino acids at the C-terminus. uPAR consists of three domains ranging in size from 81 to 87 amino acids, namely, D1, D2 and D3, which are connected by short linker regions. **(B)** The 3D structure of uPAR, with domains colored as in part(Protein Data Bank identifier 3BT2). **(C)** PAI-1 and PAI-2 exhibit inhibitory action on the uPA/uPAR system. Phospholipase C cleaves and releases uPAR from the cell membrane surface to suPAR, and uPAR cleavage results in hydrolysis of the specific SMB-binding site between D1 and D2 in the uPAR structure. Consequently, there are three different structural forms of suPAR: the complete D1+D2+D3 structure, the D2+D3 structure and the free D1 fragment.

uPAR, also known as CD-87, was discovered by Vassalli et al. in 1985 7. uPAR is a cysteine-rich glycosylated single-chain protein with a relative molecular weight of 50 kD-60 kD 8. uPAR encodes a protein of 335 amino acids comprising 22 amino acids (secreted signal peptides) at the N-terminus and 30 amino acids at the C-terminus, which is bound to the cell membrane via a glycosyl phosphatidyl inositol (GPI) anchor 9 (Fig. 1A). As reported, uPAR consists of three domains ranging in size from 81 to 87 amino acids, namely D1, D2 and D3 10, which are connected by short linker regions 11, 12 (Fig. 1B). The D1 block binds to urokinase-type plasminogen activator (uPA), the D3 region anchors uPAR to the membrane surface via a GPI, and the D2 sector joins the D1 and D3 sectors together. The N-terminus of the arginine-glycine-aspartic acid (RGD) or somatomedin-B (SMB) structure binds to the hydrophobic chamber of uPAR 13, 14 (Fig. 1B).

Transmembrane glycerophosphodiesterase GDE3, as a GPI-specific phospholipase C, cleaves and releases uPAR from the cell membrane surface to produce the soluble type of uPAR (suPAR) 9, 15, 16. suPAR contains the ligand binding sites of uPAR and is present in plasma, urine, blood, serum and cerebrospinal fluid 17. uPAR cleavage results in the hydrolysis of the specific SMB-binding site between D1 and D2 in the uPAR structure 18. Consequently, there are three different structural forms of suPAR: the complete D1+D2+D3 structure, the D2+D3 structure and the free D1 fragment 11, 12, 19-21 (Fig. 1C).

## The uPA/uPAR system

The uPA/uPAR system is composed of uPA, uPAR, plasminogen activator inhibitor-1 (PAI-1), endogenous plasminogen activator inhibitor-2 (PAI-2) and plasminogen 4, 15. PAI-1 and PAI-2 exhibit inhibitory action on the uPA/uPAR system (Fig 1C). The trimer complex formed by the binding of uPA and uPAR with PAI-1 can be recognized by lipoprotein receptor-related protein and endocytosed into cells. The uPA system regulates the interaction between cells and the ECM through proteolytic cascade reaction and further regulates cell signal transduction 22. As ligands of uPAR, uPA and vitronectin can simultaneously bind to uPAR at different binding sites 20, 23. uPA is a single-chain protein with a molecular weight of 54 kD that contains an N-terminal domain with an EGF-like sequence, through which uPA can bind to the three domains of uPAR by forming a large hydrophobic cavity 24-26. Vitronectin, a viscous glycoprotein with a molecular weight of 75 kD, is widely found in blood and ECM and interacts with different kinds of ligands 27, 28.

## The role of uPAR in tumors

**Figure 2:**
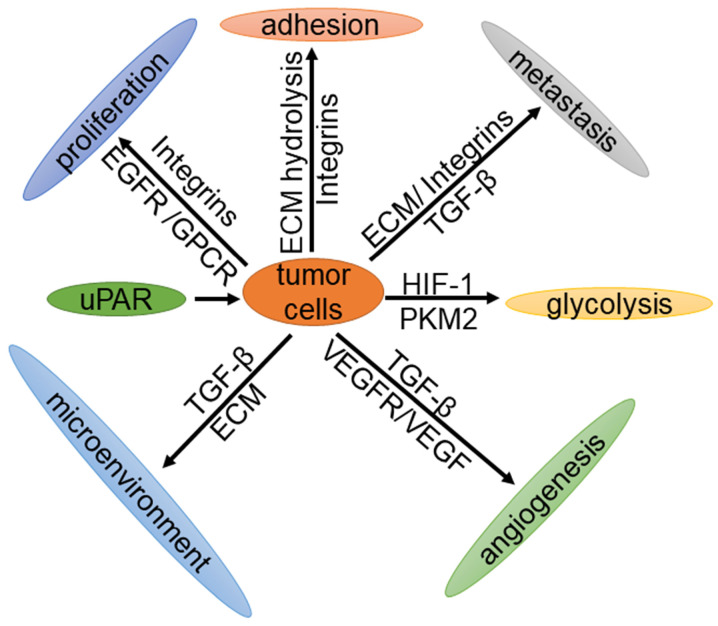
** The function of tumor cells is regulated by uPAR.** uPAR regulates the proliferation, metastasis, adhesion, glycolysis and angiogenesis of tumor cells through cell signaling, and plays an important role in the tumor microenvironment.

As early as 1991, Os-sutski et al. discovered that uPAR is closely related to cancer 29. In recent years, with the help of positron emission tomography (PET) imaging, various studies have reported that the expression levels of uPAR in patients with breast cancer, prostate cancer, bladder cancer and colorectal cancer are significantly higher than those in normal tissues 30, 31. Moreover, patients with a higher expression level of uPAR have a lower survival rate and poorer prognosis than those with lower expression 32. Knocking out the uPAR gene in mice leads to G2/M arrest, thereby inhibiting cell proliferation 33. In contrast, overexpression of the uPAR gene results in the promotion of tumor cell proliferation, migration, invasion and adhesion 4. Therefore, uPAR plays an important role in tumorigenesis and development. This review mainly describes the expression of uPAR in tumors and the important roles of the uPAR signaling pathway in tumor cell proliferation, cell adhesion, metastasis, glycolysis, the tumor microenvironment and angiogenesis (Fig. 2).

### Expression of uPAR in cancer

*uPAR* expression is elevated during inflammation and tissue remodelling and in many human cancers 4, including prostate cancer 34-36, bladder cancer 37, 38, colon cancer 39, breast cancer 40, 41, melanoma 42, brain cancer 43, lung cancer 44, renal cell carcinoma 45, liver cancer 46, 47, gastric cancer 48, 49, ovarian cancer 50, 51, head and neck cancer 52, cervical cancer 53 and pancreatic cancer 54. Sustained high expression of uPAR is associated with the growth and metastasis of cancer cells 55, 56. Moreover, uPA is also highly expressed in invasive tumors 57. The uPA/uPAR interaction can promote the expression of oncogenes and cell proliferation, eventually leading to the development of tumors 58. Knockout of the uPAR gene in tumor cells with the CRISPR/Cas9 system results in the inhibition of cell proliferation, migration and invasion 59. A decrease in uPAR expression on the cell surface mitigates the development of hallmarks of cancer caused by PIK3CA and KRas mutations in colorectal cancer 60. By interacting with uPA and IGF1R, uPAR is able to enhance the malignant potential of triple-negative breast cancer 41. More importantly, high expression of uPAR is closely related to a poor prognosis 61. In addition, studies have demonstrated that the decrease in the suPAR concentration after resection in patients with colorectal cancer 62 and pancreatic cancer 54 is associated with reduced mortality risk. Therefore, the expression level of uPAR can be assessed as a marker of tumor malignancy 30, 59, 63.

### Regulatory network of uPAR in tumor cell proliferation

**Figure 3:**
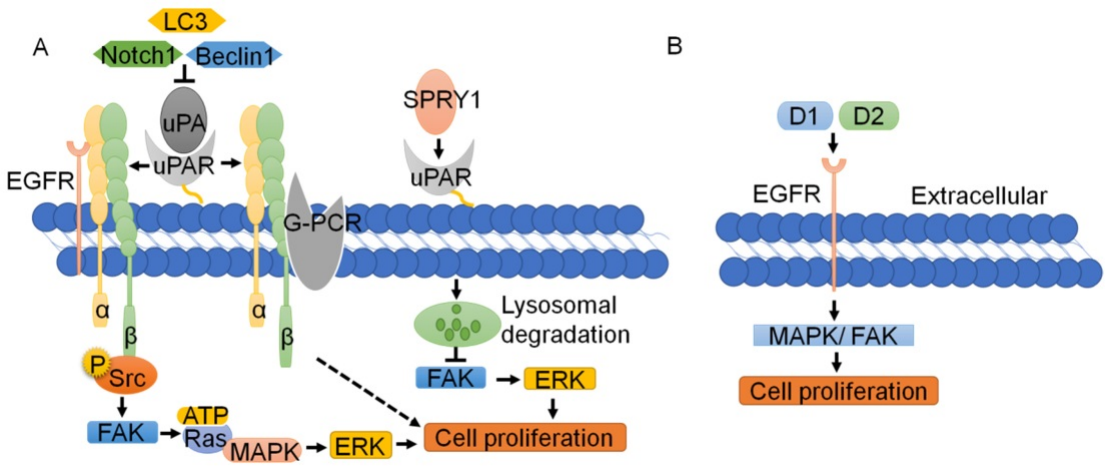
** Function and regulation of uPAR in tumor cell proliferation. (A)** The interaction of the uPA-uPAR-α5β1 integrin complex with EGFR enables the Src homology 3 domain (SH3) in the intracellular domain of integrin α5β1; this leads to the activation of FAK, which results in Ras activation and MAPK expression. The uPA-uPAR-α5β1 integrin complex can bind to GPCR to transmit signals and promote tumor cell proliferation. SPRY1 can interact with uPAR and promote its lysosomal-mediated degradation, resulting in inhibition of the activation of the FAK and ERK pathways, which suppresses the tumor proliferation induced by uPAR. **(B)** D1 and D2 of uPAR are crucial for EGFR activation, which is as effective as EGF in promoting MAPK and FAK, and cell proliferation.

Since uPAR lacks transmembrane or intracellular domains, it needs to interact with transmembrane receptors and complexes to trigger downstream signaling and promote tumor cell proliferation 4. Recently, Wang K et al. 59 and Semina EV et al. 64 knocked out uPAR with the CRISPR/Cas9 system, successfully resulting in suppression of human cancer cell proliferation. Silencing uPAR can inhibit the expression of the MMP2, MMP9 and P-ERK proteins in oral and tongue squamous cell carcinoma and attenuate cell proliferation 65. Research shows that the uPA-uPAR-α5β1 integrin complex can bind to G-protein-coupled receptors (GPCRs) to transmit signals and promote tumor cell proliferation 66. The interaction of the uPA-uPAR-α5β1 integrin complex with EGFR enables the phosphorylation of Tyr397 and the Src homology 3 domain (SH3) in the intracellular domain of integrin α5β1; this leads to the activation of focal adhesion kinase (FAK, also known as PTK2) 67-69, which results in the activation of Ras and the expression of mitogen-activated protein kinase (MAPK). uPAR can also transactivate EGFR, mediating the uPAR-initiated mitogenic signal 70, 71 (Fig. 3A). The D2A motif in domain 2, which is as effective as EGF, can promote phosphorylation of EGFR and activation of the MAPK signaling pathway, thus facilitating cell proliferation 71. D1 of uPAR is crucial for EGFR activation, and FAK links integrin and EGFR signaling. Inhibition of EGFR kinase blocks uPAR-induced ERK signaling, implicating EGFR as an important effector of the pathway 69 (Fig. 3B).

Other signaling pathways are also involved in the proliferation mediated by uPAR. The Notch pathway is a highly conserved cellular signaling system that regulates the differentiation of a variety of cells and plays important roles in carcinogenesis. As reported, silencing Notch1 can inhibit the expression of uPA and its receptor uPAR, thus inhibiting the proliferation of cancer cells 72. SPRY1, an inhibitor of the Ras-MAPK pathway, can interact with uPAR and promote its lysosomal-mediated degradation, resulting in inhibition of the activation of the FAK and ERK pathways, which suppresses the tumor proliferation induced by uPAR 73-75. Knockdown of LC3 and Beclin-1 leads to inhibition of uPAR/integrin-β1/Src signaling pathways, thereby suppressing cancer cell proliferation and colony formation 76. Loss of uPAR inhibits the PI3K/AKT pathway, while downregulation of uPAR leads to upregulation of P-ERK and forces cells to use the ERK pathway as an alternative pathway for growth and survival 77 (Fig. 3A). In short, uPAR typically binds to cell membrane surface proteins, such as integrin, EGFR and GPCR to promote cell proliferation, whereas binding of uPAR by its inhibitors leads to lysosome-mediated degradation of the uPAR, thus repressing of uPAR on the proliferation of tumor cells.

### Effect of the uPAR signaling pathway on tumor cell adhesion

**Figure 4:**
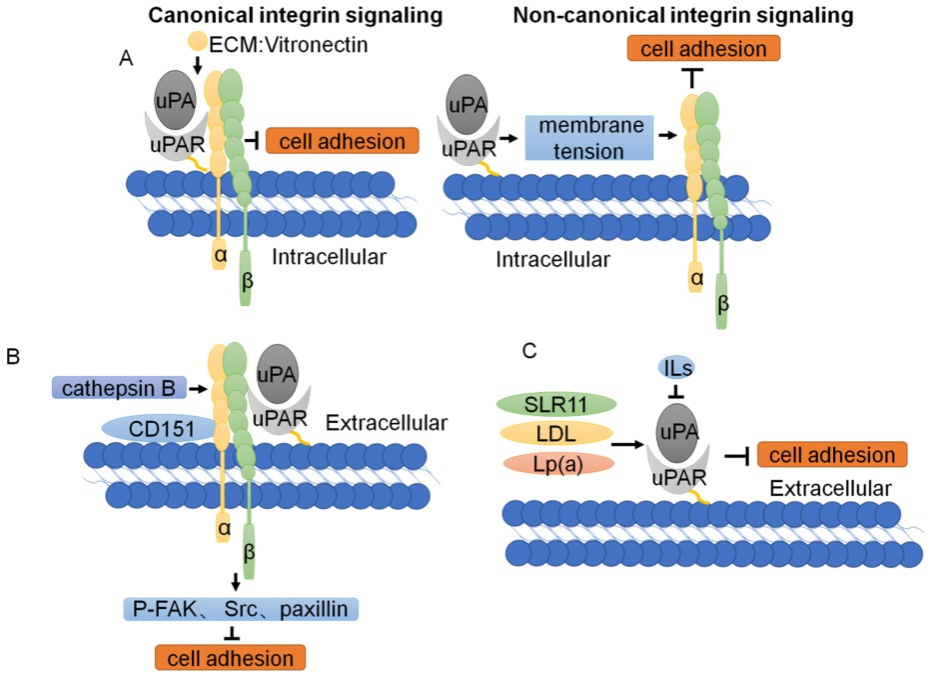
** Function and regulation of uPAR in tumor cell adhesion. (A)** Two modes of uPAR signal regulation. In canonical signaling, integrins engage the specific ligands in the ECM. In non-canonical integrin, uPAR-mediated cell adhesion, through the plasma membrane, transmits a mechanical stimulus to the integrin that signals independently of ECM binding. **(B)** The downstream signaling cascade of uPAR/CD151/α3β1 integrin shows that phosphorylation of FAK, Src and paxillin is reduced with knockdown of cathepsin B, uPAR, and CD151. **(C)** Other cellular proteins regulate tumor cell adhesion through uPAR.

In addition to the regulation of cell proliferation by uPAR described above, uPAR also regulates cell adhesion 77, 78. Changes in the physical properties, composition, expression and regulation of the ECM are considered to be abnormal signals that alter tumor cell adhesion. uPAR can regulate cell adhesion by promoting ECM proteolysis and transmitting intracellular signals 4. uPAR regulates cell adhesion by binding directly to vitronectin and by forming complexes with integrins 79. Recent studies have shown that cleavage of vitronectin by uPA displays a remarkable receptor dependence and requires concomitant binding of both uPA and vitronectin to uPAR, which induces cell adhesion 13. In contrast to canonical integrin signaling, uPAR-mediated cell adhesion to vitronectin triggers a novel type of integrin signaling that is independent of integrin-engagement. The molecular mechanism enabling the crosstalk between nonintegrin adhesion receptors and integrins is dependent on membrane tension 80 (Fig. 4A).

As mentioned above, the change in tumor cell adhesion is regulated by uPAR expression. Overexpression of uPAR can strongly upregulate MMP expression and enhance breast cancer cell adhesion 78. sLR11 regulates the hypoxia-enhanced adhesion of hematopoietic stem and progenitor cells (HSPCs) via an uPAR-mediated pathway^81^. LDL and Lp(a) lipoproteins increase the expression of uPA and uPAR on monocytes, affecting plasmin generation and monocyte adhesion. The cytokines IL-4, IL-10 and IL-13 induce a decrease in uPAR expression and lead to a change in tumor cell adhesion 82 (Fig. 4C). Studies on the downstream signaling cascade of uPAR/CD151/integrin α3β1 have shown that phosphorylation of FAK, Src, and paxillin and expression of the adaptor cytoskeletal proteins talin and vinculin are reduced with knockdown of cathepsin B, uPAR, and CD151 83 (Fig. 4B). Collectively, these data demonstrate that uPAR regulates tumor adhesion through complex mechanisms.

### Effects of the uPAR signaling pathway on tumor metastasis

uPAR is widely expressed on the surface of endothelial cells, fibroblasts and a variety of malignant tumor cells and exerts functions in cancer cell migration and tumor metastasis 14. As reported, uPAR regulates malignant tumors through integrins on breast cancer 84 and pancreatic ductal adenocarcinoma cells 85. Different studies have stressed that the regulatory effects of uPAR on tumor metastasis through other signaling pathways, such as those related to the ECM, integrins, and TGF-β1.

#### ECM

**Figure 5:**
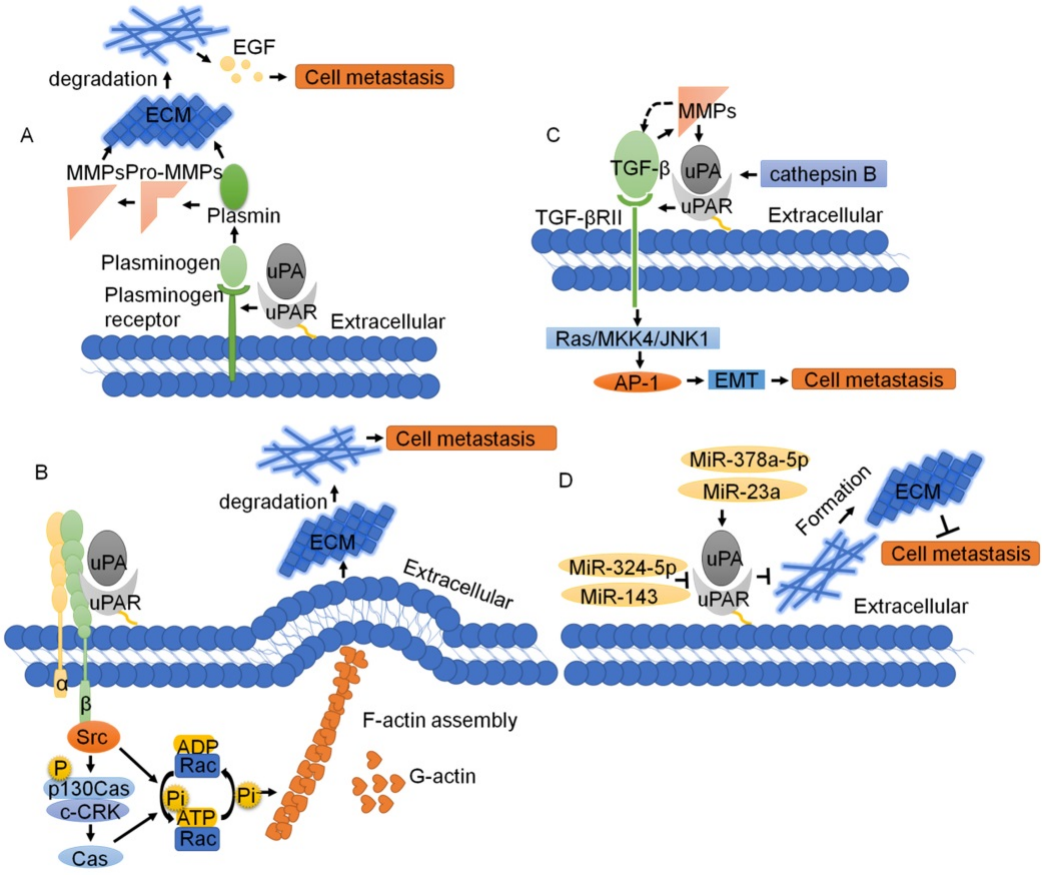
** The regulatory network of uPAR through the ECM, integrin, TGF-β and noncoding RNA in tumor migration. (A)** uPA binds to uPAR, and the inactive pro-uPA precursor is transformed into active uPA. Then uPA cleaves inactive plasminogen into active plasmin, which further cleaves and activates downstream MMPs. The fibrinolytic proteases and MMPs formed after activation will hydrolyze ECM and release active EGF, which promotes tumor invasion and metastasis. **(B)** Integrin and uPA/uPAR form the structure of the uPA-uPAR integrin complex signal, and drive the activation of GTPase Rac actin assembly, the cell wall of actin protrusions extends forward, and pericytes outside the cytoplasmic membrane protein (pericyclic protein) decomposition are eliminated outside the ECM barrier membrane. **(C)** TGF-β1 induces epithelial-mesenchymal transition (EMT) with an associated upregulation of uPA, uPAR, cathepsin B and MMP-9. TGF-β activates the Ras/MKK4/JNK1 signaling cascade, leading to the induction of AP-1 activity, which promotes cell migration. **(D)** MicroRNAs regulate uPAR-induced ECM formation and protein degradation, which play an important role in cancer cell metastasis.

Since uPAR lacks transmembrane and intracellular domains 4, it needs to interact with transmembrane receptors, such as ECM receptors and integrins, to activate intracellular signals. The ECM is required for cell movement and is a physical barrier to cell movement. Cell migration often involves the decomposition of ECM proteins 86. uPAR activates a variety of intracellular signaling pathways that promote cell invasion by regulating ECM proteolysis and synergistic actions with transmembrane receptors 4. In tumor tissues, the interaction of uPA and uPAR leads to proteolysis of the ECM through a cascade reaction. After uPA binds to uPAR, the inactive pro-uPA precursor is transformed into active uPA. Then, uPA cleaves inactive plasminogen into active plasmin, which further cleaves and activates downstream matrix metalloproteinases (MMPs) 87. The fibrinolytic proteases and MMPs formed after activation will hydrolyze ECM and release active EGF, which promotes tumor invasion and metastasis 88. uPAR can also degrade ECM through the proteasome pathway and activate MMPs to degrade ECM and activate EGF to further regulate the cell membrane ECM interaction, in addition to enhancing cell migration and signal transduction through the binding of and interaction between vitronectin and integrins 86, 89. VEGF165 interacting with its receptor VEGFR-2 rapidly induces pro-uPA activation that is dependent on a change in integrin affinity, activation of MMP-2 and pro-uPA being bound to its surface receptor uPAR 90. Taken together, uPAR is an important ECM proteolysis protein that regulates the interaction between cells and the ECM as well as cell migration 4 (Fig. 5A).

#### Integrins

Integrins are important cell adhesion receptors and play substantial roles in the progression of tumor metastasis 91. uPAR and integrins form stable complexes that both inhibit native integrin adhesive function and promote adhesion to vitronectin via a ligand binding site on uPAR 92. The uPAR and integrin α5β1 interaction promotes tumor cell migration. uPAR is required to activate integrin α5β3 in podocytes, promoting cell motility and activating the small GTPases Cdc42 and Rac1. Blockade of integrin α5β3 reduces podocyte motility *in vitro* and lowers proteinuria in mice 93. The uPA-uPAR-integrin α5β1 complex drives activation of the GTPase Rac and actin assembly. Actin protrusions from the cell wall extend forward, and pericytes outside the cytoplasmic membrane protein undergo a decomposition of pericyclic proteins, which eliminates the ECM barrier outside the cell membrane and membrane processes 94. The glycolytic enzyme alpha-enolase (ENO1) also acts as a plasminogen receptor, controls integrin α5β3 expression and upregulates pancreatic cancer invasion, and metastasis 85. The major downstream uPAR/integrin signaling (especially β1 and β3) involve activation of Src, PI3K/AKT, and MEK/ERK1-2 pathways 26. Furthermore, uPAR cooperates with integrin complexes containing integrin β3 to drive formation of the p130Cas-CrkII signaling complex and activation of Rac, resulting in a Rac-driven elongated-mesenchymal morphology, cell motility and invasion 95. uPAR interaction with vitronectin initiates a p130Cas/Rac-dependent signaling pathway, leading to actin reorganization and increased cell motility 96. Activated Rac can also stimulate actin polymerization, leading to the assembly of filamentous myosin, and ultimately stimulate membrane processes, leading to cell migration and invasion 96. In addition, integrin-uPAR signaling can lead to the phosphorylation of Fos-related antigen-1 (FRA-1), promoting the invasion of breast cancer cells 84 (Fig. 5B).

#### TGF-β and EMT

Tumor cell metastasis typically requires activation of TGF-β1 to control physiological processes 97 TGF-β signaling through mitogen-activated protein kinase, c-Jun-NH2-kinase, p38, PI3K, and G-proteins may be responsible for some of the oncogenic effects that occur in tumor cell migration and invasion 98. TGF-β1 induces invasion in malignant meningioma cells with an associated upregulation of uPA, uPAR, cathepsin B and MMP-9, and activation of intracellular signals of the H-RAS, ERK/PI3K, xIAP and MAPK pathways 99-101. In addition, TGF-β, MMPs and the uPA/uPAR system can induce epithelial-mesenchymal transition (EMT) in cancer cells 102, 103. Interestingly, TGF-β can induce MMPs expression, and MMPs can in turn activate TGF-β, promoting EMT in cancer cells 104. uPA/uPAR expression induces EMT in tumor cells by mediating TGF-β, resulting in tumor progression and metastasis 103, 105. Furthermore, TGF-βRII is required for TGF-β activation of JNK1 and the resulting upregulation of uPAR expression. TGF-β activates the Ras/MKK4/JNK1 signaling cascade, leading to induction of AP-1 activity, which, in turn, up-regulates uPAR expression 106 (Fig. 5C). In addition, as mentioned above, uPAR expression is closely related to EMT. Recent studies have shown that uPAR upregulation in melanoma cells exposed to mesenchymal stem cell (MSC)-medium drives TGFβ-mediated EMT 107. The transcription factor Forkhead box M1 (FOXM1) promote cancer EMT and metastasis by enhancing uPAR gene transcription 108, while uPAR downregulation inhibits cancer EMT and dysregulation EMT biomarker proteins 64, 103, 107. TGF-β-induced uPA expression is human telomerase reverse transcriptase (hTERT)-dependent, and a positive association exists between hTERT and uPA 101. Taken together, it is clear that both TGF-β and uPA/uPAR collaborate in the induction of cancer-associated EMT.

#### Non coding RNA

MicroRNAs are small, noncoding single-stranded RNAs that negatively regulate gene expression at the posttranscriptional level. MicroRNAs can inhibit the expression of uPAR directly and indirectly in a variety of cancer types 34. Targeted delivery of antisense-miR-21 and antisense-miR-10b coloaded in uPAR-targeted polymer nanoparticles (NPs)-treated mice show a substantial reduction in tumor growth 109. As reported, miR-378a-5p and miR-23a promote tumor cell metastasis by upregulating the expression of uPAR 110, 111. However, miR-324-5p, miR-193b and miR-143 can inhibit the expression of uPA and uPAR, thus inhibiting the migration and invasion of cancer cells 112-114. Recently studies show that miR-200s regulate ECM remodeling, which trigger tumor cell invasion 115. Taken together, uPAR is regulated by microRNAs to exert ECM remodeling, which plays an important role in the metastasis of cancer cells (Fig. 5D).

#### Other factors

Studies have also revealed that uPAR can interact with formyl peptide receptors (FPRs) to promote cell migration 116. The uPAR88-92 sequence can interact with FPR1, and inhibition of uPAR/FPR1 crosstalk may be useful for the treatment of metastatic epithelial ovarian cancer (EOC) 51, 116. Moreover, the S90P and S90E substitutions in the uPAR protein can cause upregulation and downregulation of cell migration, respectively, by mediating agonist-triggered activation and internalization of FPR1 117, thus inhibiting tumor metastasis 116. In addition, uPAR can enhance the metastasis and invasion induced by Ras mutations in tumor cells 118. In human AGS gastric cancer cells, uPAR can be stimulated by prostaglandin E2 via the EP2 receptor-dependent Src/EGFR/JNK1/2, Erk1/2/AP-1, Src/EGFR/JNK1/2, and Erk1/2/NF-κB signaling pathways, thereby promoting tumor metastasis 119. As a co-receptor, uPAR is recycled on the cell surface and redistributed to the invasive side of cancer cells, further enhancing the migration and invasion abilities of cancer cells 4.

Different studies have stressed that uPAR has contributory effects on tumor metastasis through other signaling pathways. Silencing of uPAR inhibits the invasion and migration of oral tongue squamous cell carcinoma cells by regulating the expression of MMP2, MMP9 and p-ERK 65. PDZ-binding kinase (PBK) can bind directly to the core region of the uPAR promoter through ETV4 to regulate the metastasis of hepatocellular carcinoma 120. In bladder cancer, uPAR can regulate the mammalian target of rapamycin complex (mTORC) signaling pathway. uPAR silencing inhibits AKT phosphorylation at Ser473, inhibiting cell migration and invasion 37.

### Regulatory network of uPAR in glycolysis

**Figure 6:**
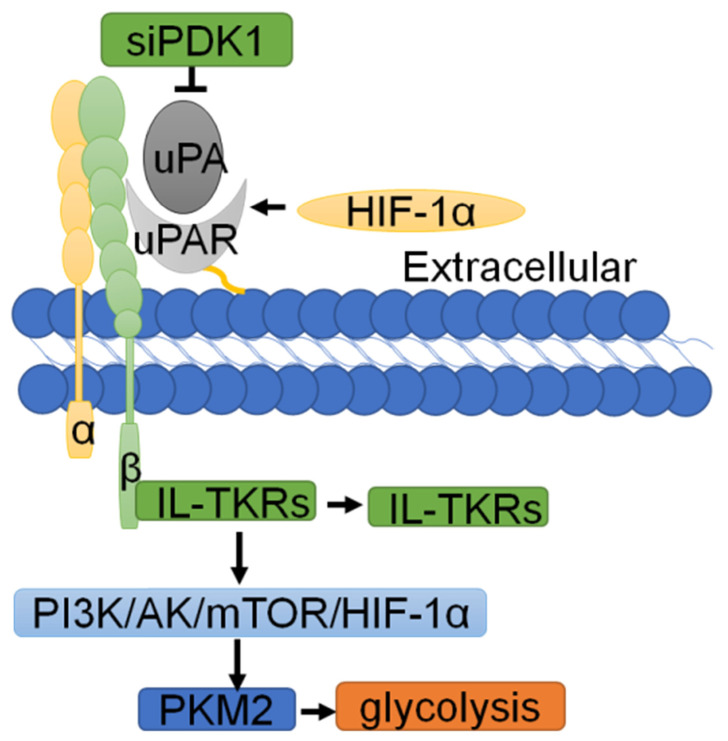
** The regulatory network of uPAR in glycolysis.** Hypoxia can enhance the expression of endogenous uPAR in a HIF-1α dependent manner. Inhibition of uPAR with siRNA or uncoupling of uPAR from integrin-linked tyrosine receptors (IL-TKRs) inhibits the PI3K/AKT/mTOR/HIF-1α signaling pathway, resulting in impaired glucose uptake and a reduction in PKM2 and other glycolytic enzymes, thereby controlling the metabolism of cancer cells.

Normal cells rely on mitochondrial oxidative phosphorylation to produce ATP, while cancer cells, which are not affected by the partial pressure of oxygen, are able to gain energy via glycolysis with the stimulation of hypoxia-inducible factor (HIF-1α) 6. As early as 1997, Anichini E and colleagues discovered that uPAR plays an important role in glycolysis 121. The interaction of uPA with uPAR rapidly induces the activation of glucose transporters. In recent years, studies have found that hypoxia can enhance the expression of endogenous uPAR in a HIF-1α-dependent manner 122. As reported, activation of HIF-1α can upregulate uPAR expression and activate its associated signals 123, 124, while inhibition of HIF-1α gene expression can downregulate the mRNA and protein levels of Upar 125. Mechanistically, inhibition of uPAR with siRNA or uncoupling of uPAR from integrin-linked tyrosine receptors (IL-TKRs) will inhibit the PI3K/AKT/mTOR/HIF1α signaling pathway, resulting in impaired glucose uptake and a reduction of pyruvate kinase-2 (PKM2) and other glycolytic enzymes, thereby controlling the metabolism of cancer cells 6. In addition, phosphoinositide-dependent protein kinase-1 (PDK1) can inhibit glycolysis in cancer cells 126. Downregulation of PDK1 through the use of siRNAs targeting uPAR leads to the downregulation of downstream P-Akt 127, 128 (Fig. 6).

### Regulatory network of uPAR in the tumor microenvironment

A study focused on the microenvironment of colorectal tumors found that uPAR is expressed in macrophages, neovascular endothelial cells and myofibroblasts, and its expression is negatively correlated with survival rates 32. Another study also showed that uPAR contributes to vascular permeability, resulting in changes in the inflammatory microenvironment in ovarian cancer 129. In breast cancer, researchers have established a mathematical model of cancer recurrence focusing on monitoring of tissue biomarkers, including markers in the plasminogen system, and found that only the serum concentration of uPAR in cancer patients was positively correlated with cancer recurrence 130. The interaction of uPA with uPAR activates a network of interconnected signaling pathways and induces and activates the tumor microenvironment regulatory factor TGF-β 100, which in turn promotes the expression of uPA and thus forms a positive feedback loop 131. TGF-β function through proteolytic degradation of the ECM and regulates the expression of several MMPs and uPA/uPAR in cancer cells, thus contributing to tumor malignancy 103, 132. uPAR also controls the expression of the tumor microenvironment regulator IL-4 in cancer cells by activating the ERK1/2 pathway 133, 134.

### Role of uPAR in tumor-associated angiogenesis

Angiogenesis plays a critical role in physiological and tumor pathological processes 135. The blood vessels allow blood to reach all parts of the growing tumor mass, providing nutrients and oxygen, and allow invading tumor cells to reach distant sites for colonizatione. New blood vessels can sprout from pre-existing angiogenesis or can form by endothelial progenitor cells (EPCs) 136. During angiogenesis, endothelial cells (ECs) degrade basement membrane, migrate through the ECM, proliferate and organize in new vessels, which can include locally recruited EPCs. uPAR activation consequent to the binding of uPA can be regarded as an “angiogenic switch” 137. uPAR focuses on the proteolytic activity of uPA on the endothelial cell surface, thus promoting angiogenesis in a protease-dependent manner. In endothelial cells, uPAR interacts with VEGFR2, which mediates VEGF signaling and promotes angiogenesis 138. VEGF165, the major angiogenic growth factor that initiates angiogenesis, requires coordinated proteolytic degradation of extracellular matrix provided by the uPA/uPAR system and regulation of cell migration provided by integrin-matrix interaction 66, 139. Evidence shows that VEGF165, VEGF-E, FGF-2, EGF and HGF induced PI3K-dependent activation of pro-uPA when bind to uPAR, which leads to an increase in cell surface fibrinolytic activity 140. Thus, uPAR represents a central mediator of growth factor-induced endothelial cell migration.

The amoeboid and mesenchymal types of invasiveness are two modes of interchangeable migration in cancer cells. A recent study showed that a role of the uPAR-integrin-actin axis in the regulation of amoeboid angiogenesis. uPAR is indispensable for ECs and ECFCs to perform efficient amoeboid angiogenesis 141. uPAR is also functionally important in fostering angiogenesis in EPCs 142 and ECFCs 143 upon recruitment in caveolar-lipid rafts. Gangliosides and uPAR typically partition into specialized membrane microdomains called lipid-rafts. The cell membrane enrichment with exogenous GM1 ganglioside is pro-angiogenic, with the opposite effect of cell membranes enriched with GM3 ganglioside. Following GM1 exogenous addition, the GM3 compartment is depleted of uPAR which is recruited within caveolar rafts thereby triggering angiogenesis 142. Endothelial uPAR is also thought to provide a regulatory mechanism in angiogenesis. The proangiogenic role of uPAR in ECFCs, depends on the integrity of caveolae and the presence of full-length uPAR in specialized membrane invaginations. Inhibition of uPAR expression promoted caveolae disruption. VEGF promoted the accumulation of uPAR in ECFCs caveolae in its undegraded form. VEGF-dependent ERK phosphorylation required integrity of caveolae as well as caveolar uPAR expression. Interestingly, overexpression of matrix metalloproteinase-12 (MMP-12) blocks angiogenesis by cleavage of endothelial uPAR 144, which impairs angiogenesis in SSc 145. MMP12- dependent uPAR cleavage results into the loss of invasion properties and angiogenesis 146. VEGF activity depends on inhibition of ECFC MMP12 production, which impairs MMP12-dependent uPAR truncation. MMP12 overexpression in ECFCs inhibits vascularization *in vitro* and *in vivo* 143. Angiogenesis and tumor promotion are active in late stages of tumor progression by TGF-β. Evidence shows that TGF-β upregulates the expression of uPAR to reguate pro-angiogenic activity in human normal dermal MVEC 147. Inhibition of GDF5 in TGFß-stimulated ECs impairs TGFß-dependent uPAR overproduction, impairing angiogenesis 148. Exosomes is a new vesicular lipid transporter that is involved in various pathophysiologies. uPAR-expressing melanoma exosomes promote angiogenesis by VE-cadherin, EGFR and uPAR overexpression and increase ERK1/2 signaling in endothelial cells 149, 150. Tumor suppressor phosphatase and tensin homologue (PTEN) expression in endothelial cells is downregulated by uPAR to activate the PI3K/Akt pathway and support angiogenesis 151. Mice deficient in uPAR provided an opportunity to assess the role of uPAR during angiogenesis *in vivo*. In uPAR(-/-) mice, dermal fibrosis is paralleled by endothelial cell apoptosis and a severe loss of microvessels 152. Similarly, tumor growth of subcutaneously injected murine prostate cancer cells is significantly retarded in uPAR-deficient mice compared with wild-type mice 153. In conclusion, uPAR plays an important role in angiogenesis *in vivo* and *in vitro*.

## uPAR and cancer therapy

### uPAR and chemoradiotherapy

Chemoradiotherapy (CRT) plus surgery for locally advanced cancer has recently become the standard therapeutic strategy and has a significant survival benefit compared with surgery alone 154-156. Some studies have attempted to accurately assess CRT responses with different diagnostic approaches, but the results have mostly been unsatisfactory. Therefore, reliable and effective biomarkers to predict the sensitivity and response of advanced tumors to CRT are urgently needed to promote individualized treatment. A recent study investigated the profiles of cytokines related to EGF and uPAR in 68 esophageal squamous cell carcinoma (ESCC) patients. The data indicated that upregulation of uPAR- and EGF-related cytokines after CRT is associated with poor progression-free survival and shortened survival 156. The levels of EGF and uPAR for CRT in serum are reliable and predictive biomarkers for survival in ESCC patients 156. However, the expression of uPAR is dramatically upregulated after CRT 157, and recent results suggest that PAI-1 but not uPA and uPAR might have prognostic value for patients with advanced non-small-cell lung cancer (NSCLC) undergoing radiotherapy 158. Therefore, the response of individual tumors to CRT is highly variable.

### uPAR and targeted therapy

The uPAR system regulates cell proliferation, adhesion, invasion, and migration as well as glycolysis and the microenvironment. PAI-1 mediates the endocytosis of uPAR and blocks its biological function, thus inhibiting tumor development 159. Therefore, uPAR can be used as a marker for cancer prognosis and diagnosis and is an attractive therapeutic target 4, 160. Quercetin has been proven to induce antimetastatic effects in gastric cancer cells by suppressing the uPA/uPAR system via modulation of various associated pathways, including the NF-κb, PKC-δ, ERK1/2, and AMPKα pathways, indicating that uPAR may be a potential target for the treatment of gastric cancer 161. The plant flavonoid 2',3,4',5,7‑pentahydroxyflavone can effectively inhibit the expression of uPA and uPAR and inhibit TPA-induced metastasis of human breast cancer cells through the Akt/GSK-3β/C-FOS pathway 162. Moreover, apigenin plays an anti-invasive role by mediating the (ERK1/2, JNK)/AP-1 and (ERK1/2, JNK)/NF-κB signaling pathways to inhibit the expression of uPAR 163. As uPAR and FPR1 are both involved in tumor progression, an effective cell migration peptide inhibitor (Ac-d-Tyr-d-Arg-AIB-d-Arg-NH) has been synthesized to inhibit the interaction of uPAR and FPR1 to suppress migration and angiogenesis 116, 164. Another study also stressed that inhibitors of uPAR84-95/FPR1 crosstalk may be useful for the treatment of metastatic melanoma 165. 2G10, a recombinant antibody that binds to uPAR to form a stable complex and can block the interaction of uPA-uPAR, is effective in a xenotransplantation model of highly aggressive, triple-negative breast cancer (TNBC) 166. Anti-uPAR small molecules that specifically inhibit the uPAR-vitronectin interaction can inhibit cell adhesion and migration, representing a novel tool for NSCLC and colorectal cancer patients carrying Ras mutations 118.

### uPAR and immunotherapy

**Figure 7:**
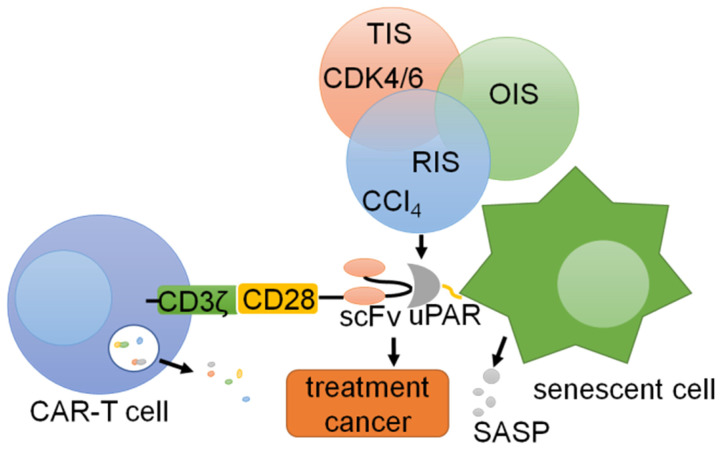
** CAR-T cells targeting uPAR can be utilized for cells.** uPAR is identified as a common upregulated marker in senescent cells in three different models: therapy-induced senescence (TIS), oncogene-induced senescence (OIS), and replication-induced senescence (RIS). Senolytic CAR T cells were generated by introducing anti-mouse uPAR scFv linked to human CD28 costimulatory and CD3ζ signaling domains, resulting in T cell activation and degranulation.

Chimeric antigen receptors (CARs) are synthetic receptors that can alter the specificity and other functions of T cells 167, 168. Preventing the occurrence of various diseases caused by the accumulation of cellular senescence is important for immunotherapy of tumors and other diseases 169. Thus, CAR-T cells that counter aging-associated changes exhibit broad therapeutic potential 167, 170. uPAR is widely expressed on the surface of senescent cells, and uPAR-targeted CAR-T cells can eliminate senescent cells *in vitro* and *in vivo* 167. In T cells, the CAR includes an extracellular uPAR-specific ligand binding domain (scFv), an intracellular costimulatory domain (from molecules such as CD28 or 4-1BB) and a CD3ζ T cell activation domain, which is activated by the binding of uPAR, leading to the activation and granule shedding of intracellular T cells 171, 172. suPAR can be used as a plasma biomarker to evaluate the anti-aging activity of CAR-T cells *in vivo* 173. Therefore, uPAR can be used as a target for CAR-T cell therapy in cancers 174 (Fig. 7). These works provide promising preliminary evidence that uPAR-directed CAR-T cells effectively target senescent cells and show that this CAR T-cell treatment has a measurable impact on disease states in immunocompetent hosts.

Antibodies against uPAR can inhibit pericellular hydrolysis, thus blocking the downstream signaling pathways activated by uPAR as well as tumor growth and metastasis 175, 176. huATN-658, a humanized anti-uPAR antibody, can significantly decrease tumor cell proliferation and metastasis 177. Leukocyte immunoglobulin- like receptor B4 (LILRB4) is an inhibitory immune receptor that is more highly expressed in monocytic AML cells than in normal monocytes 178. A recent study revealed that LILRB4 can regulate different signaling pathways, including the uPAR pathway, suppress T cell activity and promote the proliferation of leukemia cells 178. Therefore, the detection or targeting of uPAR in immunotherapy may also be of interest.

### uPAR and cell drug resistance

Cell drug resistance can occur over time in the treatment of cancer and results in the weakening of drug effects, which is one of the causes of cancer-related death 179. To reduce drug resistance, combination drug therapy has become an important method for effective treatment of cancer 180. Exosomes are extracellular vesicles ranging in size from 40 nm to 100 nm that are often secreted by tumor cells and multiple stromal cells in the tumor microenvironment, and they can enhance drug resistance 128, 181. The expression of uPAR is an important reason for cetuximab resistance in patients with oral squamous cell carcinoma, and combination therapy with resveratrol, which can inhibit the expression of uPAR, may provide an attractive means for treating these patients 182. Knocking out the uPAR gene via the CRISPR/Cas9 system can reduce the resistance of tumor cells to 5-FU, cisplatin, docetaxel and Adriamycin 59. BRAF inhibitor (BRAF-I) therapy for melanoma patients is initially highly effective, but drug resistance greatly limits its application. A recent study demonstrated that uPAR knockdown in combination with vemurafenib administration can inhibit melanoma cell proliferation by decreasing the phosphorylation of AKT and ERK1/2, and overexpression of uPAR results in reduced sensitivity to BRAF inhibition 183. Researchers have also found that an anti-uPAR antibody (HuATN-658) combined with bisphosphonate zoledronic acid (Zometa) can inhibit breast cancer growth and bone lesions by targeting uPAR 177.

## Conclusions and prospects

Tumorigenesis and progression via the uPAR signaling pathway have emerged as hot topics in the field of cancer research. uPAR is a GPI type multifunctional receptor that mainly binds to ligand molecules released by ECM hydrolysis, such as uPA and vitronectin, and then combines with integrins and G-PCR on the cell membrane to transmit the signal intracellularly 184. This review provides an overview of emerging data, from basic research as well as cancer therapy, highlighting the evolving role of uPAR in tumor progression. It is currently believed that uPAR expression plays an important role in tumorigenicity, and high endogenous uPAR levels are associated with tumor proliferation, advanced metastatic cancers, and glycolytic capacity 185. uPAR has also been implicated in the angiogenesis of several solid and hematologic malignancies 186. uPAR is aberrantly expressed through activation of signaling pathways by genetic alterations, oncogenes, transcription factors, and microenvironmental influences. Additionally, various therapeutic strategies have emerged in preclinical animal testing and clinical trials to inhibit the functions of uPAR in cancer therapy. However, the clear molecular mechanism need to be further investigated in immune escape.

Targeted uPAR immunotherapy has not achieved the desired effects in the treatment of various types of cancers. One reason for this inconsistent and poor response may be related to individual differences among patients as well as tumor heterogeneity within a single patient. Therefore, the search for targeted drugs that can inhibit the binding of uPAR and uPAR target proteins as well as other membrane proteins has become extremely important 187. Some small molecules and antibodies that can either suppress the expression of uPAR or block the interaction between uPAR and related membrane proteins are able to inhibit the development of tumors. A combination of resveratrol and cetuximab inhibits the expression of uPAR and has been used to treat cancer 184. Moreover, uPAR-targeted CAR-T cells can eliminate senescent cells *in vitro* and *in vivo* 167, indicating the broad therapeutic potential of uPAR in immune therapy. Coronavirus disease 2019 (COVID-19) is characterized by suppressed lung fibrinolysis. Recent studies have shown that uPA can regulate alveolar type 2-mediated re-alveologenesis 188. The expression of suPAR is highly correlated with the characteristics of COVID-19 patients. Thus, studies of the uPA/uPAR system are helpful for identifying drugs to prevent or even treat COVID-19 189. As such, further analyses of immune therapy and disruption of the interactions between uPAR and its coreceptors represent an attractive strategy for targeting aggressive malignancies.

## Abbreviations

uPA: Urokinase-type plasminogen activator receptor
uPAR: Urokinase-type plasminogen activator
suPAR: soluble type of uPAR
ECM: Extracellular matrix
GPI: Glycosyl phosphatidyl inositol
RGD: Arginine-glycine-aspartic acid
SMB: Somatomedin-B
PAI-1: Plasminogen activator inhibitor-1
PAI-2: Plasminogen activator inhibitor-2
MMPs: Mitochondrial Membrane Potentials
PET: Positron emission tomography
G-PCR: G-protein-coupled receptors
EGF: Epidermal Growth Factor
EGFR: Epidermal Growth Factor Receptor
FAK: Focal adhesion kinase
MAPK: Mitogen activator protein kinase
ERK: Extracellular signal-regulated kinase
TGF-β: Transforming growth factor-β
VEGF: Vascular endothelial growth factor
ENO1: Enzyme alpha-enolase1
EMT: Epithelial-mesenchymal transition
FPRs: Formyl peptide receptors
EOC: Epithelial ovarian cancer
mTORC: mammalian target of rapamycin complex
HIF-1α: Hypoxia-inducible factor-1α
IL-TKRs: Integrin-linked tyrosine receptors
KM2: Pyruvate kinase-2
PDK1: phosphoinositide-dependent protein kinase-1
CRT: Chemoradiotherapy
ESCC: Esophageal squamous cell carcinoma
NSCLC: Non-small-cell lung cancer
TNBC: Triple-negative breast cancer
CARs: Chimeric antigen receptors
LILRB4: Leukocyte immunoglobulin-like receptor B4
Zometa: Zoledronic acid
COVID-19: Coronavirus disease 2019
EPCs: endothelial progenitor cells
ECs: endothelial cells
PTEN: phosphatase and tensin homologue
hTERT: human telomerase reverse transcriptase
FOXM1: Forkhead box M1
